# Identification of *Wolbachia* new strains from *Aedes aegypti* mosquitoes, the vector of dengue fever in Jeddah Province

**DOI:** 10.1186/s12866-023-03010-9

**Published:** 2023-10-06

**Authors:** E. Sharawi Somia, Ihsan Ullah, Hanan S. Alyahya, Jazem A. Mahyoub

**Affiliations:** 1 Department of Biology Sciences, Faculty of Sciences, King Abdulaziz University, Jeddah, Saudi Arabia; 2 IBB University, Ibb, Republic of Yemen

**Keywords:** *Wolbachia* Sp., *Wsp*, *Aedes* sp., Molecular marker, 16S rRNA

## Abstract

*Wolbachia* are endosymbiotic bacteria found within many arthropods, including insects. A variety of benefits are provided by these bacteria to human and insect hosts, including protection from viruses and parasites and the ability to kill males. In this study, *Wolbachia* was identified in *Aedes aegypti* present in Jeddah, Saudi Arabia. A population of mosquitoes was collected from eight different areas, processed, and tested for *Wolbachia* using 16 S rRNA specific to *Wolbachia* bacteria and *Wolbachia* surface protein (*wsp*) under optimized PCR conditions. In five ecologically diverse sites to determine *Wolbachia* prevalence, we identified eleven diverse novel resident *Wolbachia* strains within *Ae. Aegypti* for the first time in Jeddah, Saudi Arabia. Future studies to evaluate the possible use of *Wolbachia* as a control agent in *Aedes* sp. in Saudi Arabia are necessary. *Wolbachia* prevalence rates and strain characterization through Sanger sequencing with multilocus sequence typing (MLST) and phylogenetic analysis revealed significant diversity. In developing biocontrol strategies, it is beneficial to consider the implications of resident *Wolbachia* strains.

**Supplementary Information:**

The online version contains supplementary material available at 10.1186/s12866-023-03010-9.

## Introduction

Mosquitoes belong to the order Diptera (Culicidae), and they are the most alarming insect threat to human health. Mosquitoes kill millions of people around the world by transmitting diseases. Mosquitoes and humans inhabit the same ecological area, clearly showing that there are more *Aedes aegypti* mosquitoes than any other insect group [1, 2]. The females of most species have piercing and sucking mouth parts, and it appears that for their eggs to mature properly, they must consume mammalian blood at least once. Males eat fruit and plant juices despite having proboscis or beaks that cannot be used for piercing [3]. An effective mosquito vector of a human illness is one that readily uses humans as a source of blood meals and persists in high densities close to humans across a wide geographic area [4, 5]. Mosquitoes are important arbovirus vectors and carriers of serious human diseases such as Zika virus, dengue DHF, Chikungunya, and yellow fever [6].

Most dengue fever control strategies involve chemical insecticides and community movement to reduce breeding places for mosquitoes during the larval, pupal, and adult stages [7, 8]. As a result of the use of these chemical insecticides, several undesirable effects have been observed, including chemical resistance, toxic effects on nontarget organisms, and health risks to humans and the environment [9, 10].

*Wolbachia* are endosymbiotic bacteria that live within the cells of many arthropods, including insects such as mosquitoes [11]. These bacteria provide a variety of benefits to their insect hosts, such as resistance to viruses and protection against parasitoids [12]. These bacteria are thought to be the most common endosymbiont found in arthropods and are especially prevalent in insects [13]. There are many different strains of *Wolbachia*, each of which is specific to a particular host species, and it is estimated that *Aedes aegypti* is infected with *Wolbachia* in up to 75% of wild populations [14].

The first evidence of *Wolbachia* infection in mosquito species was discovered in 1991 when researchers studying filarial nematodes in humans noticed that some patients had unusually high numbers of *Wolbachia* in their blood cells [15]. They suspected that the bacterium was causing cytoplasmic incompatibility, where infected females produce eggs that lack sperm and therefore cannot develop into viable offspring [15]. In 1999, scientists working on the yellow fever virus discovered that *Wolbachia* could also cause cytoplasmic male sterility (CMS) in mosquitoes [16]. This meant that males carrying *Wolbachia* would not be able to reproduce because they lacked sperm [17]. Additionally, *Wolbachia pipientis* (*wPip*) was the first strain discovered in the mosquito *Culex pipiens*. In addition, *wAlb* has been isolated from *Aedes albopictus*. In Australia, the Eliminate Dengue Programme approved the transfer of *Wolbachia* into mosquitoes to control dengue virus transmission. Many public health organizations use *Wolbachia* to control dengue virus and other arthropod-borne viruses. [14]. With the increasing problem of pesticide resistance spreading in a number of cities and towns, including Jeddah, Makkah, Jizan and Al-Madinah [18], substantial efforts are being made to develop environmentally friendly strategies to reduce mosquito populations or limit their potential to transmit disease.

This study aims to identify different *Wolbachia* strains from laboratory and field strains of *Ae. aegypti* mosquitoes from Jeddah city and to determine the prevalence of *Wolbachia* strains in the area.

## Materials and methods

### Collection of Insects

#### Colony establishment (lab-strain)

A random group of *Ae. aegypti* population from the areas belonging to Al-Safa district was selected. The Jeddah Municipality, in cooperation with King Abdulaziz City for Science and Technology, had previously released large numbers of mosquitoes carrying the bacteria *Wolbachia* for the purpose of gathering adults of *Ae. aegypti* mosquitoes. The goal here was to ascertain the transmission of bacteria from parents to offspring by detecting the bacteria in the resulting offspring. The criteria used for sample size determination in the experiment were the district area, the number of cases of dengue fever, and the population density of the vector of dengue fever. The BG-Sentinel Mosquito Trap was used, and the Global Positioning System (GPS) coordinates of these sites were determined by projecting the coordinates of the sites covered by the study on the map of Jeddah Governorate using the Geographical Information System (GIS). Once per week, at least 12 h before sunset, traps were placed at each site and collected the next morning. The density of the released lab strains was approximately 500,000, according to the reports of the Jeddah Municipality. The contents of each trap were emptied into cages for rearing adult mosquitoes, which were cubic cages composed of metal frames of equal dimensions (30 × 30 × 30 cm). A special code number was given for each trap. To obtain eggs, mosquitoes were fed a blood meal using the Hemotek Membrane Feeding System. A small plastic cup with water was placed in the middle of the breeding cage after 4–6 days of blood feeding. Mosquitoes were reared in the laboratory at a temperature of 27 ± 1 °C, a relative humidity of 70 ± 5%, 14 h of light and 10 h of darkness until the exit of the adults (first generation). Adult males and females were taken 10 days after they left the pupal stage. Samples were then taken from each box, and the presence of *Wolbachia* bacteria in their tissues was determined using PCR separately for each sample. The positive mosquitoes were kept separate and propagated in the laboratory to obtain a laboratory strain carrying the bacteria. according to Table 1.

### Collection of insects (field-strain)

**Fig. 1:**
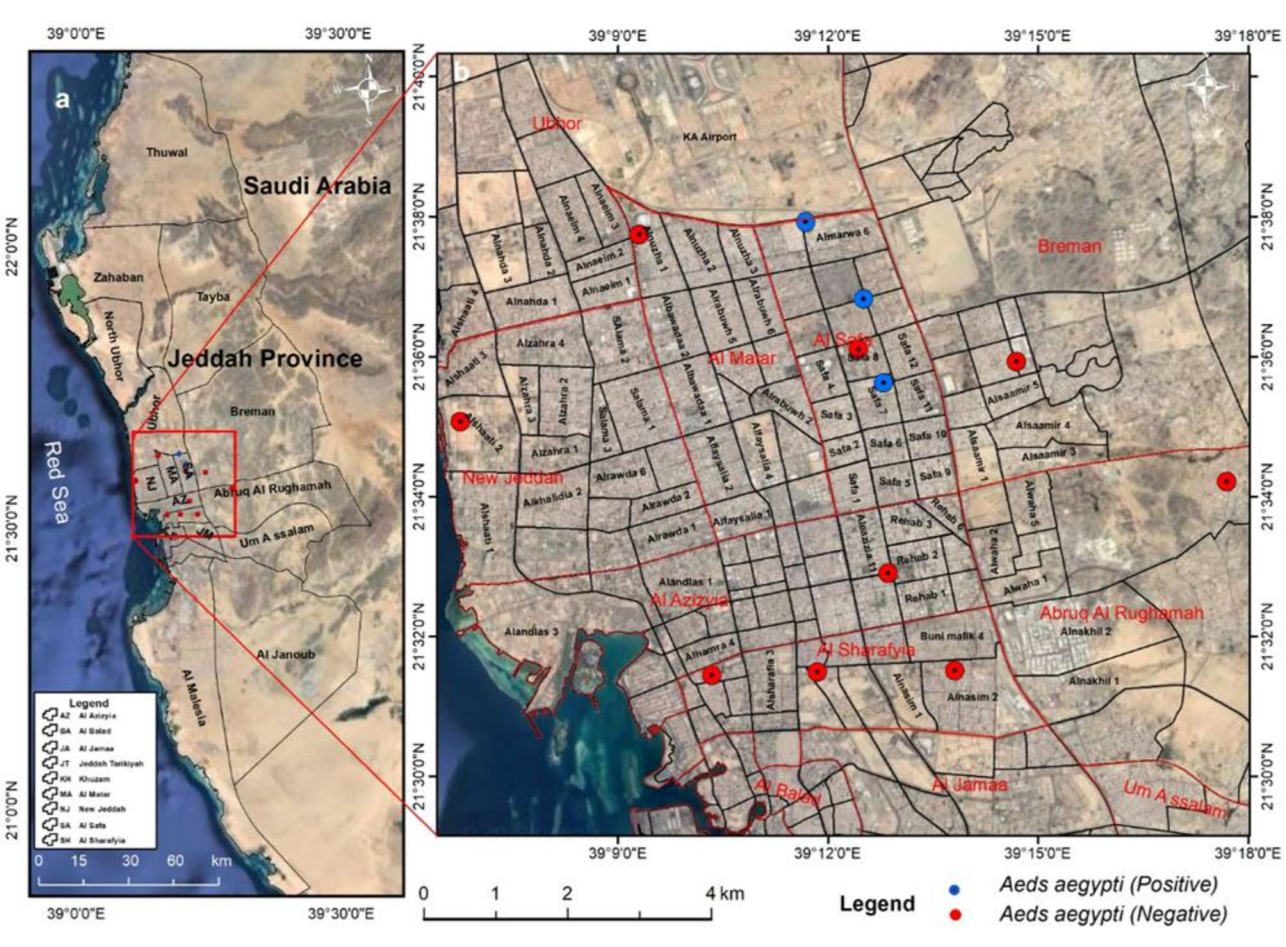
Map showing mosquitoes collected from 22 different states in Jeddah Province

To isolate bacteria from field strains, traps were distributed in several random areas according to Fig. 1; Table 2, and the insects belonging to the species *Ae. aegypti* were separated and preserved in 70% alcohol until the detection of bacteria, and each sample was labeled with a separate code number.

**Table 1:** Distribution of the adult *Ae. aegypti* sampling sites (n = 22)

ID	District	N	E
**1**	Alsafa	21.6321000	39.1948100
**2**	Alsafa	21.6137200	39.2085900
**3**	Alsafa	21.6016300	39.2073500
**4**	Alsalamah	21.5937600	39.2133500
**5**	Almatar	21.6290100	39.1551800
**6**	Almatar	21.6290100	39.1551800
**7**	Almatar	21.6290100	39.1551800
**8**	Biriman	21.5987500	39.2449400
**9**	Biriman	21.5987500	39.2449400
**10**	Biriman	21.5987500	39.2449400
**11**	Jeddah aljadida	21.5844400	39.1126400
**12**	Jeddah aljadida	21.5844400	39.1126400
**13**	Jeddah aljadida	21.5844400	39.1126400
**14**	Abraq alrughama	21.5701900	39.2949700
**15**	Abraq alrughama ‘	21.5701900	39.2949700
**16**	Abraq alrughama	21.5701900	39.2949700
**17**	Alsharafia	21.5251700	39.2302500
**18**	Alsharafia	21.5248000	39.1975600
**19**	Alsharafia	21.5240200	39.1724600
**20**	Aleazizia	21.5483700	39.2143800
**21**	Aleazizia	21.5483700	39.2143800
**22**	Aleazizia	21.5483700	39.2143800

### DNA extraction

A DNeasy Blood and Tissue Kit (Qiagen, Hilden, Germany) was used to extract genomic DNA. The insects were first homogenized (bead homogenizer) and then digested overnight at 56 °C following the manufacturer’s specifications. Total DNA was eluted into 200 µL and stored at − 40 °C.

### Polymerase chain reaction (PCR)

The PCR technique was applied according to [19]. The primer sequences were *wsp* 81 F for detecting *Wolbachia* infection using two molecular markers, *wsp* and 16 S rRNA (5′-TGGTCCAATAAGTGA TGAAGAAAC-3′), and *wsp* 691R (5′- AAAAATTAAA CGCTACTCCA-3′) for the *wsp* marker, and the 16 S *Wolbachia*-specific primers were *Wolbachia*_F (16S_WspecF) (5′-CATACCTATTCGAA GGGATAG-3′) and *Wolbachia*_R (16S_WspecR) (5′- AGCTTCGAGTGAAACCAATTC-3′). The *wsp* gene was amplified in a thermocycler. Each amplification was performed in 25 µl containing 1x GoTaq_Green Master Mix (Promega, USA), 2 µl of DNA template, and 1 µl each of forward and reverse primers (10 pmol). The amplification was performed by heating the sample at 95 °C for 5 min and 35 cycles of 94 °C for 30 s, 59 °C for 30 s and 72 °C for 45 s, followed by a final extension step of 72 °C for 10 min. In the final step, the temperature was set at 4 °C for an indefinite amount of time. The 16 S rRNA gene was amplified in 25 µl containing 1x GoTaq_Green Master Mix (Promega, USA), 2 µl of DNA template, and 1 µl each of forward and reverse primers (10 pmol). The amplification was performed by heating the sample at 95 °C for 5 min and 35 cycles of 94 °C for 30 s, 55 °C for 30 s and 72 °C for 45 s, followed by a final extension step of 72 °C for 10 min. Then, we set it to 4 °C for an indefinite amount of time. All PCR amplification experiments included negative controls (water). Two microliters of DNA amplicon were assessed using 1.5% agarose gel electrophoreses at 100 V in an electrophoresis system for 25 min in TAE buffer (40 mM Tris-acetate, 1 mM EDTA, pH 8.0). A 100 bp DNA ladder (Promega, USA) was used as a marker. The PCR product of the *wsp* gene was approximately 610 bp, while that of the 16 S rRNA gene was approximately 438 bp. To validate the PCR amplification results, the positive sample was sequenced using Sanger sequencing at Macrogen, South Korea, using forward and reverse primers for each gene.

## Results

### Resident strain density

**Fig. 2:**
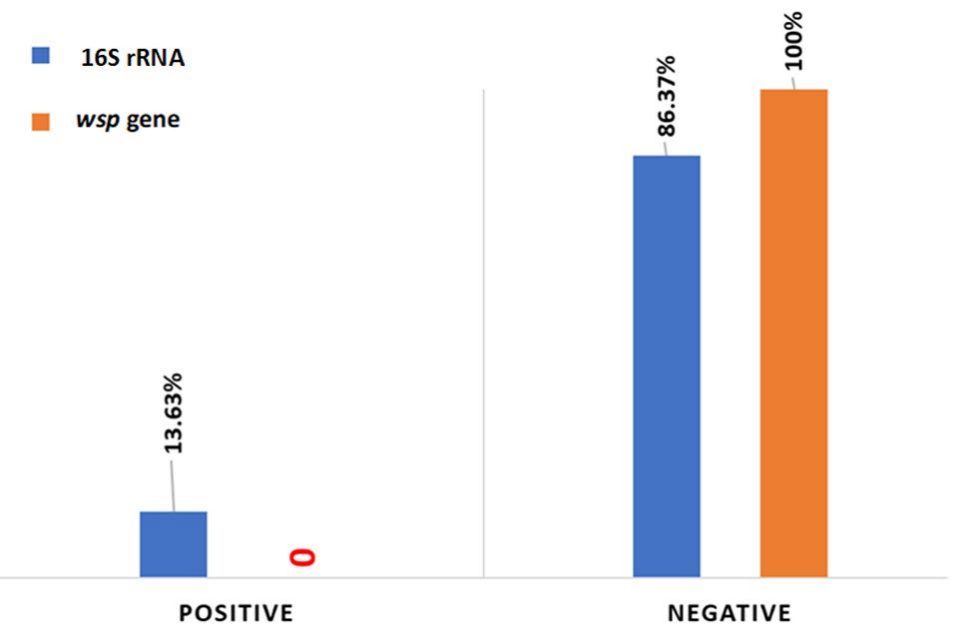
Positive and negative rates in *Ae. aegypti* using two *Wolbachia* genes (16 S rRNA and *wsp*) in a field strain

Our study investigated whether *Wolbachia* bacteria are present in *Aedes* samples collected from different areas of Jeddah city. Twelve field samples were taken for bacterial isolation and species-level identification. The results showed that three samples were positive when examining the 16 S rRNA gene, while no infected samples were found when examining the wsp gene. In this study, we found that 13.63% of the *Ae. aegypti* individuals were infected with *Wolbachia* bacteria according to the 16 S rRNA gene test and (0.0%) according to the *wsp* gene (Fig. 2; Table 2).

**Fig. 3:**
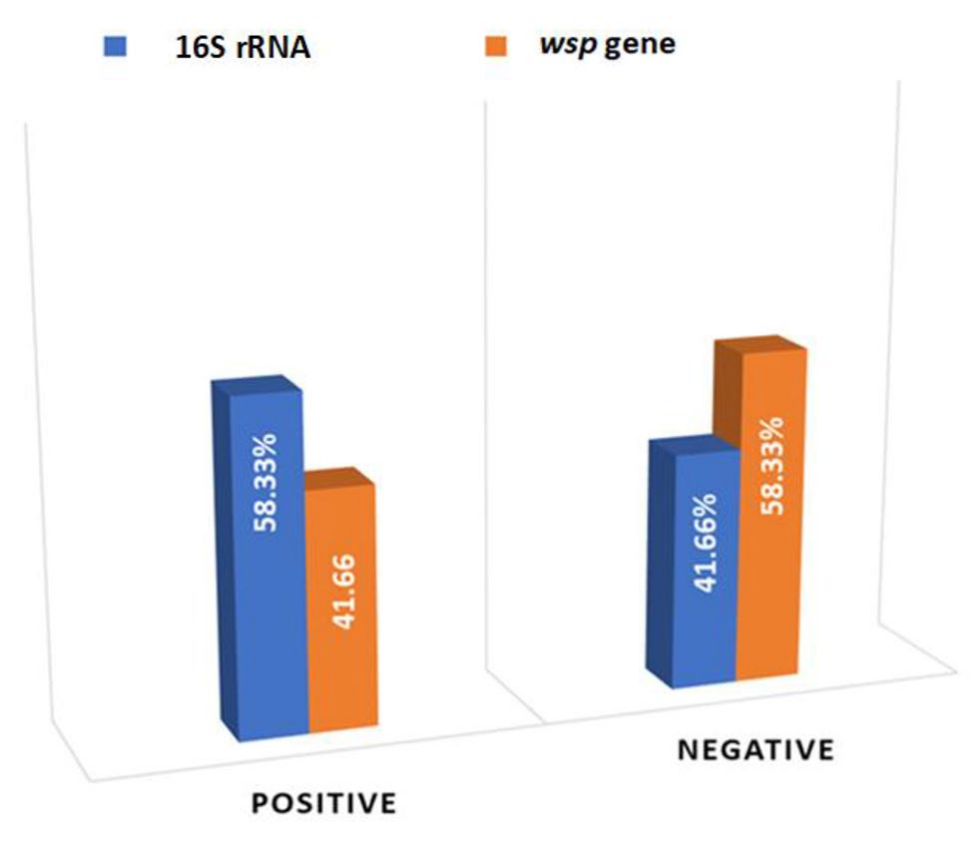
Positive and negative rates in *Ae. aegypti* using two *Wolbachia* genes (16 S rRNA and *wsp*) in a lab strain

After three generations of backcrossing between infected Ae. aegypti and noninfected *Ae. aegypti* under laboratory conditions, random samples were taken, and *Wolbachia* was detected in the tissues. In this study, twenty lab samples were taken for bacterial isolation and species-level identification. The results showed that 58.33% were positive when examining the 16 S rRNA gene, while 41% were positive when examining the *wsp* gene (Fig. 3; Table 2).

**Table 2:** A detailed summary of the results of *Wolbachia* in *Ae. aegypti* mosquitoes using the 16 S and *wsp* genes

Samples		16 S rRNA gene	*wsp* gene
Lab strains	L1	Positive	Negative
	L2	Positive	Positive
	L3	Negative	Negative
	L4	Positive	Positive
	L5	Negative	Negative
	L6	Positive	Positive
	L7	Negative	Negative
	L8	Positive	Negative
	L9	Positive	Positive
	L10	Positive	positive
	L11	Negative	Negative
	L12	Negative	Negative
Field strains	F1	Positive	Negative
	F 2	Positive	Negative
	F 3	Negative	Negative
	F 4	Positive	Negative
	F 5	Negative	Negative
	F 6	Negative	Negative
	F 7	Negative	Negative
	F 8	Negative	Negative
	F 9	Negative	Negative
	F 10	Negative	Negative
	F 11	Negative	Negative
	F 12	Negative	Negative
	F 13	Negative	Negative
	F 14	Negative	Negative
	F 15	Negative	Negative
	F 16	Negative	Negative
	F 17	Negative	Negative
	F 18	Negative	Negative
	F 19	Negative	Negative
	F 20	Negative	Negative
	F 21	Negative	Negative
	F 22	Negative	Negative

### *Wolbachia* strain typing

**Fig. 4:**
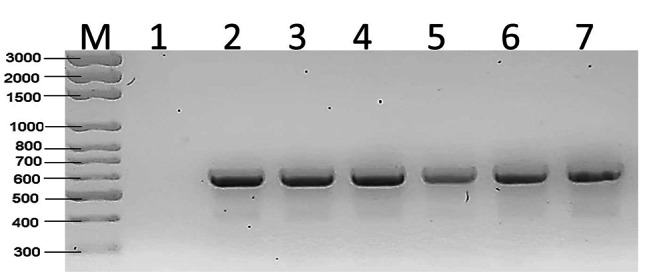
Gel electrophoresis results of the *Wolbachia* strain identification PCR assays for *Ae. aegypti* using the *wsp* gene, M: Marker, 1 negative control, sample from 2: 7

The *Wolbachia* surface protein gene (*wsp*) is a molecular marker that is used to identify the *Wolbachia* bacteria in mosquitoes by its surface protein. In this study, the *wsp* gene, which encodes the surface protein of *Wolbachia*, was sequenced. It was demonstrated through the use of *Wolbachia*-specific primers (*wsp* 81 F) that *Ae. aegypti* (n = 10) can be infected with *Wolbachia*. *Wolbachia* was detected using a specific primer, and the gene size was calculated at 600 bp (Fig. 4).

**Fig. 5:**
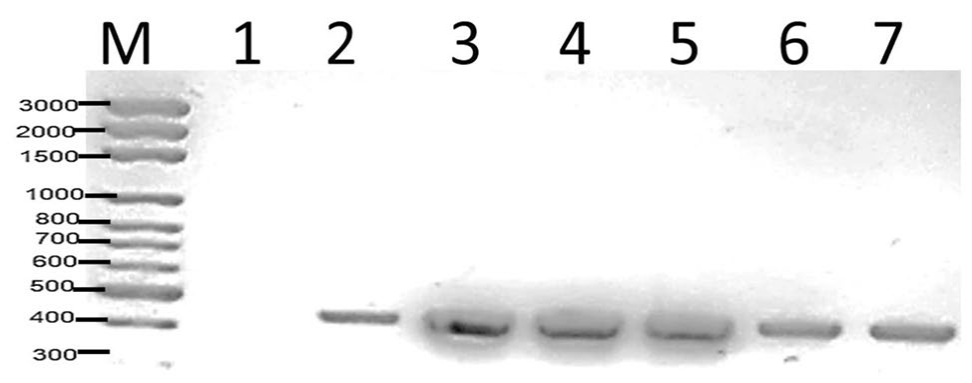
Gel electrophoresis results of the *Wolbachia* strain identification PCR assays for *Ae. aegypti* using the 16 S rRNA gene, M: Marker, 1 negative control sample from 2:7

The presence of *Wolbachia* DNA was assessed in *Ae. aegypti* mosquitoes using 16 S rRNA. *Wolbachia* DNA was extracted and amplified through PCR using 16 S rRNA markers. The Wolbachia DNA was identified in samples, and the length of the DNA was 450 bp, which was appropriate for the 16 S rRNA gene length (Fig. 5).

### Phylogenetic analysis of isolates based on 16 S rRNA

**Fig. 6:**
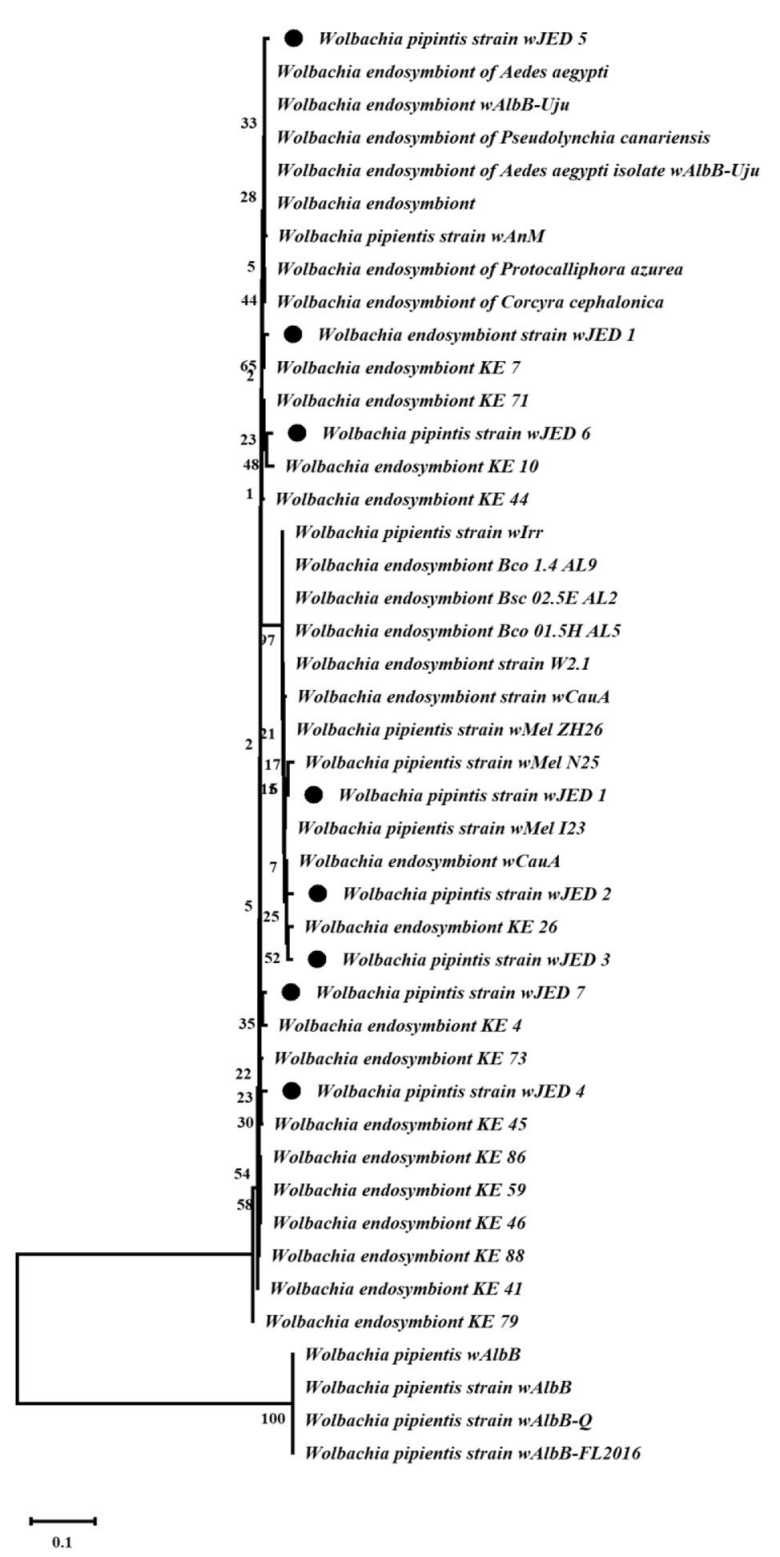
DNA sequence of *Wolbachia* was amplified using 16 S rRNA-specific primers through PCR. The sequences were searched against the NCBI nucleotide database, and the most similar match was downloaded. The neighbour-joining method was used to build the phylogenetic tree, and 1000 bootstrap values were used

Phylogenetic analysis of *Wolbachia* was carried out based on 16 S rRNA sequences obtained from mosquitoes and other reference sequences in the NCBI through BLAST. Evolutionary distances were calculated using the maximum composite likelihood method, and bootstrap support values (1000 replicates) were used to construct the phylogenetic tree. According to the phylogeny based on 16 S rRNA sequences, all the bacterial strains found in *Ae. aegypti* were *Wolbachia*, which showed a high degree of similarity (> 98%) to 16 S rRNA *Wolbachia* sequences from *endosymbionts* and *pipientis* (Fig. 6).

### Phylogenetic analysis of isolates based on the *wsp s*equence

**Fig. 7:**
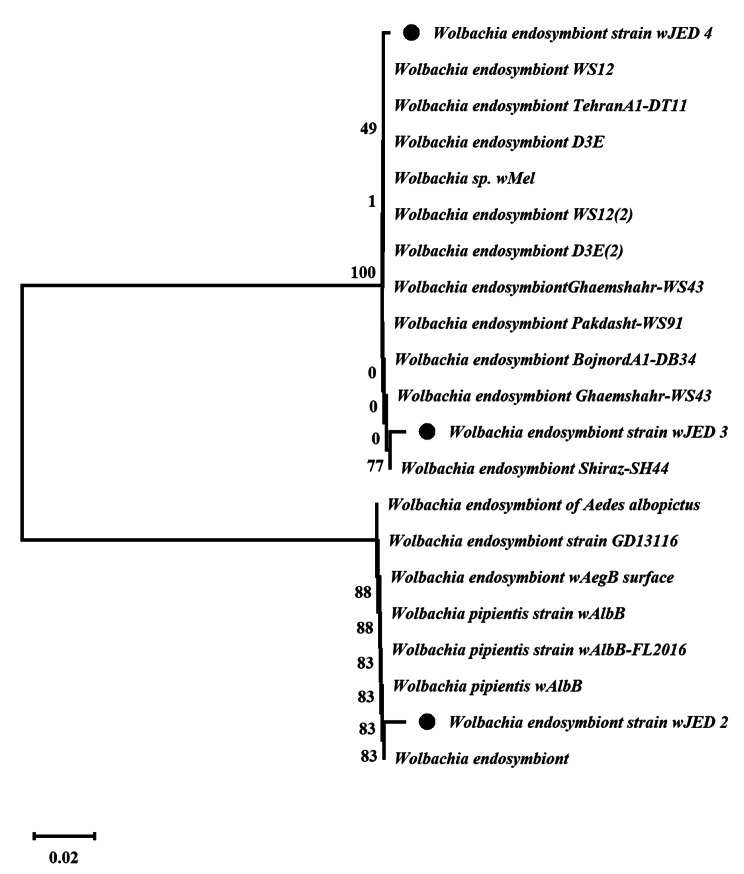
The *wsp* gene of *Wolbachia* was amplified through PCR using *wsp-*specific primers, and the sequences were searched against the NCBI nucleotide database for similar matches. Neighbour joining was used to construct a phylogenetic tree using bootstrap support values based on 1000 replicates

The following results were obtained based on phylogenetic analyses of *Wolbachia* sequences from this study and reference sequences. Bootstrap tests (1000 replicates) indicate the percentage of trees in which a specific taxon clusters together next to a branch. Evolutionary distances were calculated by maximum composite likelihood. Bootstrap values strongly supported the relationship among the isolates and *Wolbachia* strains. Phylogenetic analyses showed that *Ae. aegypti* harboured *Wolbachia* strains (Fig. 7). In the current study, 11 new resident *Wolbachia* strains were recorded for the first time in Saudi Arabia and published in the NCBI database, according to Table (3).

**Table 3:** Novel resident *Wolbachia* strains using the 16 S and *wsp* genes

Strains code	Accession number
Wolbachia_pipintis_strain_wJED_1	OQ214197
Wolbachia_pipintis_strain_wJED_2	OQ214198
Wolbachia_pipintis_strain_wJED_3	OQ214199
Wolbachia_pipintis_strain_wJED_4	OQ214200
Wolbachia_pipintis_strain_wJED_5	OQ214201
Wolbachia_pipintis_strain_wJED_6	OQ214202
Wolbachia_pipintis_strain_wJED_7	OQ215752
Wolbachia_endosymbiont_strain_wJED_1	OQ215753
Wolbachia_endosymbiont_strain_wJED_2	OQ269454
Wolbachia_endosymbiont_strain_wJED_3	OQ269455
Wolbachia_endosymbiont_strain_wJED_4	OQ269456

**Table 4:** Accession numbers and their links in NCBI database

Strains code	Accession number	Accession number in NCBI database
Wolbachia_pipintis_strain_wJED_1	OQ214197	https://www.ncbi.nlm.nih.gov/nuccore/OQ214197
Wolbachia_pipintis_strain_wJED_2	OQ214198	https://www.ncbi.nlm.nih.gov/nuccore/OQ214198
Wolbachia_pipintis_strain_wJED_3	OQ214199	https://www.ncbi.nlm.nih.gov/nuccore/OQ214199
Wolbachia_pipintis_strain_wJED_4	OQ214200	https://www.ncbi.nlm.nih.gov/nuccore/OQ214200
Wolbachia_pipintis_strain_wJED_5	OQ214201	https://www.ncbi.nlm.nih.gov/nuccore/OQ214201
Wolbachia_pipintis_strain_wJED_6	OQ214202	https://www.ncbi.nlm.nih.gov/nuccore/OQ214202
Wolbachia_pipintis_strain_wJED_7	OQ215752	https://www.ncbi.nlm.nih.gov/nuccore/OQ215752
Wolbachia_endosymbiont_strain_wJED_1	OQ215753	https://www.ncbi.nlm.nih.gov/nuccore/OQ215753
Wolbachia_endosymbiont_strain_wJED_2	OQ269454	https://www.ncbi.nlm.nih.gov/nuccore/OQ269454
Wolbachia_endosymbiont_strain_wJED_3	OQ269455	https://www.ncbi.nlm.nih.gov/nuccore/OQ269455
Wolbachia_endosymbiont_strain_wJED_4	OQ269456	https://www.ncbi.nlm.nih.gov/nuccore/OQ269456

## Discussion

*Aedes* mosquitoes were tested for the presence of *Wolbachia* bacteria to determine whether they were harmful [20]. There may still be some strains that remain undetected because of differences in tissue tropism [21]. When mosquitoes are infected with the *Wolbachia* bacterium, it can prevent diseases such as dengue fever and chikungunya from spreading [22]. Furthermore, *Wolbachia* bacteria have been shown to be toxic to other parasites, such as Plasmodium, indicating that they may also affect the transmission of other parasites [20]. Increasing temperatures reduce the density of *Wolbachia* endosymbionts in *Aedes* bacteria [23]. According to [24], low endosymbiont density in *Ae. aegypti* probably contributed to the low infection rate. In addition, studies from non-vector systems show that *Wolbachia* replication, dissemination, vertical transmission, fitness effects, and cytoplasmic incompatibility vary with temperature, and because of this wide range of thermal sensitivities, patterns of *Wolbachia*-induced transmission blocking might be strongly influenced by local environment, and unfortunately, there are no current studies showing the effect of temperature on *Wolbachia* [23]. In metabarcoding, studies found few sequences in the midgut of *Ae. aegypti*, indicating a low density of the endosymbiont [25].

Several *Wolbachia* strains have been identified that presumably influence both the normal rate of human disease transmission and the manipulation of transmission rates by *Wolbachia*-infected mosquitoes as well as the transmission rate itself [25]. *Wolbachia* strains are known to protect viruses in arboviral hosts. [20] claimed the *Wolbachia* they identified in *Ae. bromeliae* could reduce the transmission of arboviral diseases.

According to our phylogenetic analysis in the present study, the *Wolbachia* strains found in our sample of *Ae. aegypti* mosquitoes belong to *Wolbachia endosymbionts* and *Wolbachia pipientis*, respectively. Eleven residents of *Wolbachia* strains were identified and published in the NCBI database as a first record. Mosquito species with medical significance display these types of characteristics, i.e., *An. Gambiae* and *Ae. albopictus*. It remains to be confirmed whether some *Wolbachia* strains cause pathogenic effects in *Ae. aegypti* mosquitoes [15, 25]. A number of these strains have been found in dipterans, particularly mosquitoes, and have been shown to cause cytoplasmic incompatibility, male killing, and feminization. *Ae. aegypti* mosquitoes carry *Wolbachia* strains that cause these phenotypic effects, but it is unclear whether they are responsible for them [21].

To explain the clustering of mosquito-infecting strains, several explanations can be offered. There is a possibility that *Wolbachia* was acquired by horizontal transmission from a previously infected ancestral species or that *Wolbachia* was cospecified with a previous host. Compared to other taxa, horizontal transmission has been shown to occur between close relatives most commonly in horizontal transmission [26].

### Electronic supplementary material

Below is the link to the electronic supplementary material.

Supplementary Material 1

## Data Availability

The datasets generated during the current study are available in the NCBI database repository, and persistent web links or accession numbers to datasets can be found in Table 4.
