# Supplementary material for: Identification of Wolbachia new strains from Aedes aegypti mosquitoes, the vector of dengue fever in Jeddah Province
Source: BMC Microbiol. 2023 Oct 6;23:287. doi: 10.1186/s12866-023-03010-9 (PMC10557223; doi:10.1186/s12866-023-03010-9)
Supplement: Supplementary file 1 — Supplementary Material 1 [file 12866_2023_3010_MOESM1_ESM.pptx]

## Slide 1
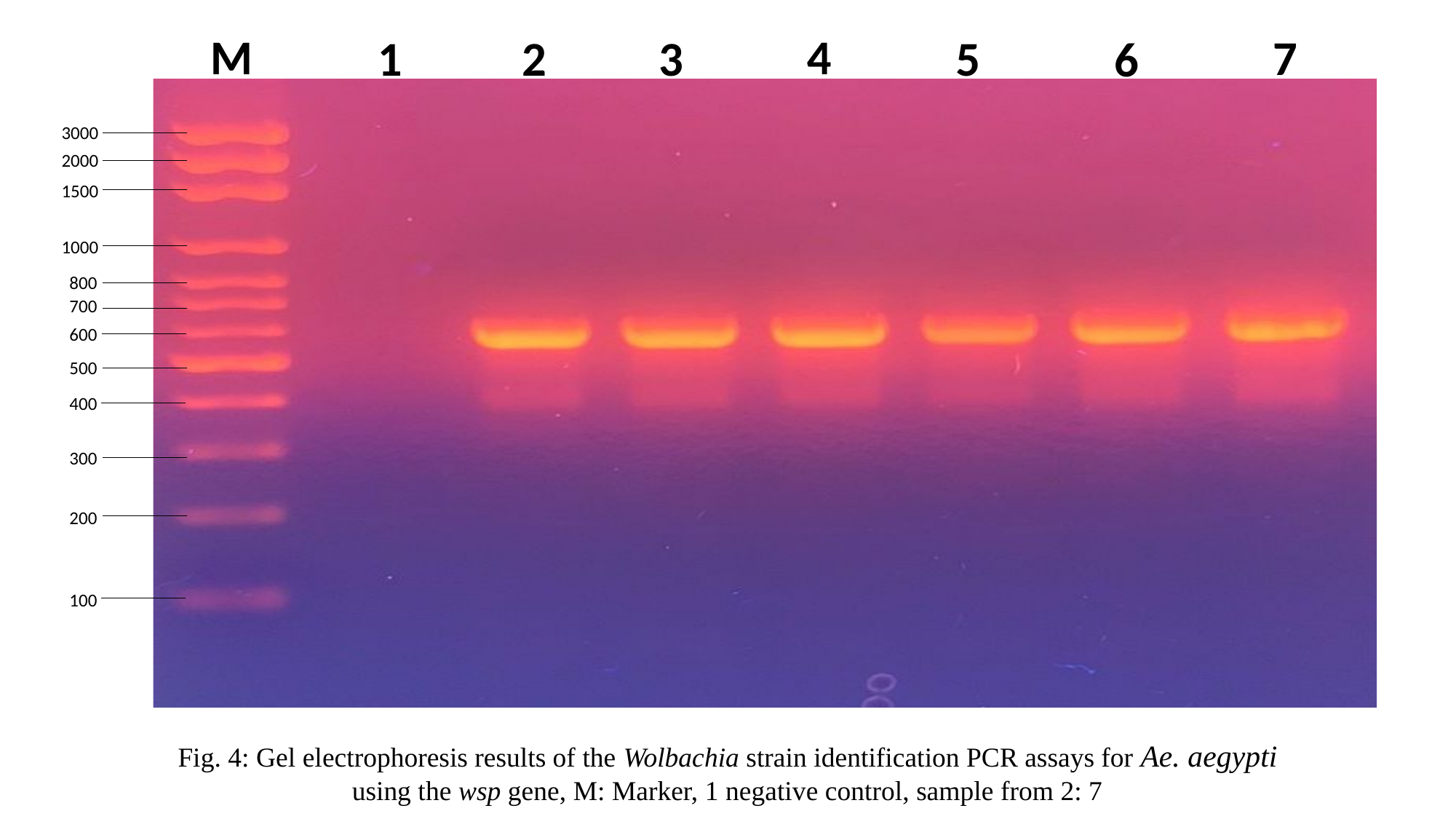

4
7
1
2
3
5
6
M
3000
2000
1500
1000
800
700
600
500
400
300
200
100
Fig. 4: Gel electrophoresis results of the Wolbachia strain identification PCR assays for Ae. aegypti using the wsp gene, M: Marker, 1 negative control, sample from 2: 7

## Slide 2
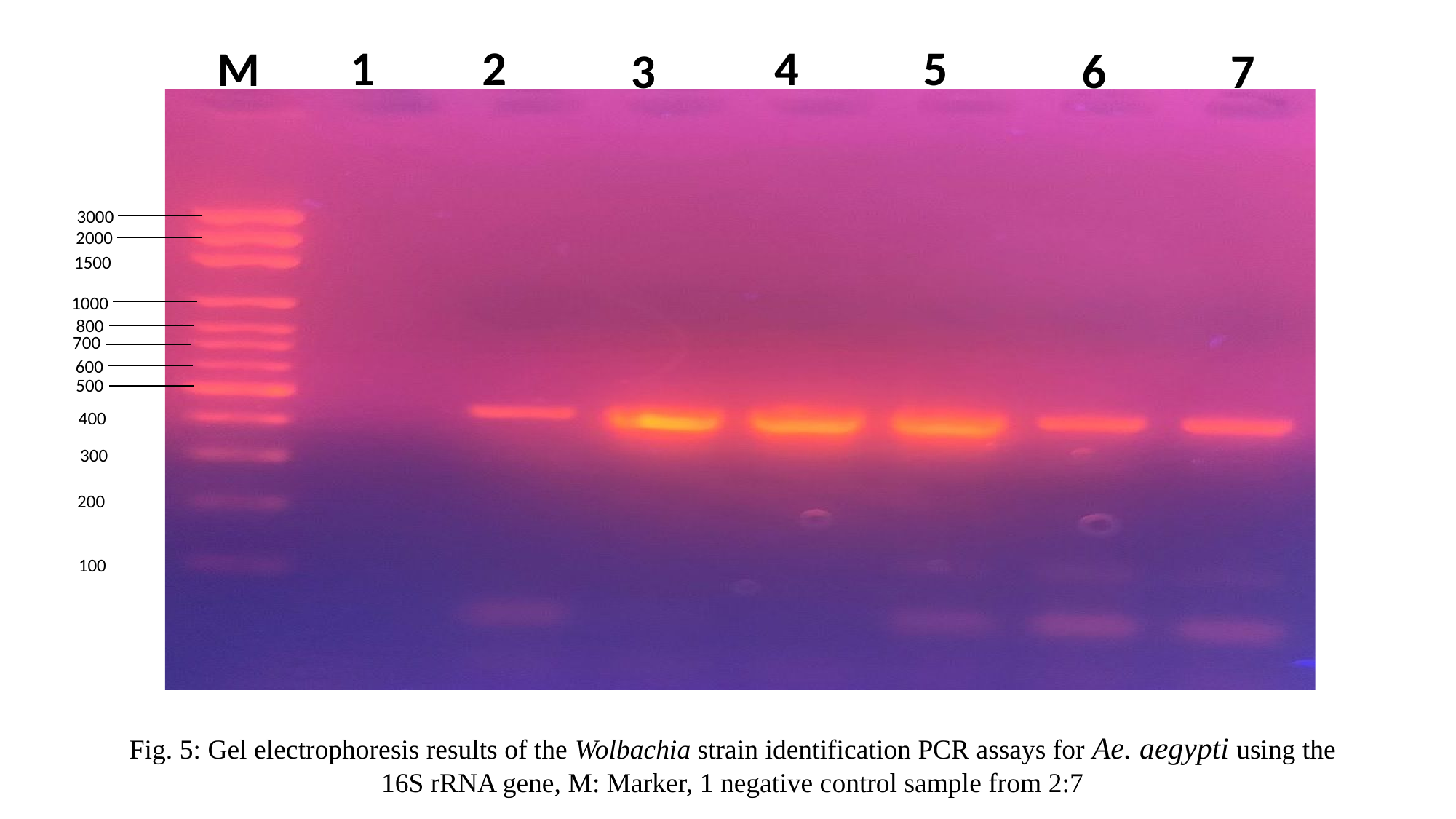

1
2
4
5
3
6
7
M
3000
2000
1500
1000
800
700
600
500
400
300
200
100
Fig. 5: Gel electrophoresis results of the Wolbachia strain identification PCR assays for Ae. aegypti using the 16S rRNA gene, M: Marker, 1 negative control sample from 2:7
